# A Case of Hernia Cerebri

**Published:** 1852-05

**Authors:** D. M. Cooper


*A Case of Hernia Cerebri successfully treated by Pressure.* Read before the St. Louis Medical Society. By D. M. Cooper, M. D.—On the 17th January, 1849, I was requested to see a boy, called Bradly, about 13 years old, who, a few days previous, had fallen from a horse, producing a compound and comminuted fracture of the cranium, a little anterior to the centre of the right parietal bone. The detached pieces of bone were removed immediately after the accident, and the patient had been under judicious treatment. When I first saw him he had slight fever; his skin was hot and rather dry; his face was flushed; the tongue was moist, however. The wound in the head was very offensive, owing to the integuments being in a sloughing condition. The boy's mind was unimpaired. I ordered the wound to be dressed with one part of the liquor of Labarraque to 4 parts of water, applied by means of lint. I also ordered the following diaphoretic:

Take Spts. Ætheris Nitrici, 1 ounce;

Liquoris Ammoniæ Acet. 3 "

M. S.—a dessert-spoonful every 2d hour.

His bowels were open, consequently I did not prescribe a cathartic.

18th. The skin moist; pulse not so frequent; face still flushed and the wound in the same condition. The fœtor had been corrected in some degree by the chloride of soda. Treatment continued.

19th. Patient doing well. Same dressing continued to the wound. A dessert-spoonful of the diaphoretic to be given every 4th hour.

24th. The patient has been progressing favorably since the 19th. He has been on very light diet and has taken the diaphoretic at intervals with an enema. The wound was clean this morning, the slough having separated during the night, bringing to view, what I apprehended, a *hernia cerebri.* After a thorough examination, I became satisfied that the protrusion was a true hernia cerebri, and not a growth from the *pia mater.* It was about the size of the half of a walnut, and not constricted at its base—there being no impaction in the orifice. In consequence of the latter circumstance, I did not dread sloughing. I placed a compress saturated in a solution of sulphate of zinc (2 grains to the ounce of water) over the cerebral protrusion and then applied a bandage lightly. The febrile symptoms having disappeared, I discontinued the diaphoretic, but left strict injunctions relative to the necessity of light diet and perfect quietude.

25th. The boy doing well, but the hernia increased in size. I continued the same dressing, increasing the pressure.

26th. The protrusion was diminished in size, and the edges of the wound in the scalp showed signs of healthy granulation.

July 10th. The treatment by pressure was continued up to this date. The hernia has receded completely within the cranial orifice, and granulation has proceeded so favorably that there is only a small circular opening in the scalp, from which healthy pus is discharged.

August 9th. The wound in the scalp has closed. There is no disposition on the part of the cerebrum to protrude; the elevation and depression of the cicatrix, however, mark the cerebral pulsations. I discontinued my visits this day, after recommending the patient to continue the application of the compress and bandage for some time, and after giving directions respecting a protective plate to be worn in future.—*St. Louis Med. and Surg. Journal.*

